# Management of Recurrent Meningiomas: State of the Art and Perspectives

**DOI:** 10.3390/cancers14163995

**Published:** 2022-08-18

**Authors:** Marco Vincenzo Corniola, Torstein R. Meling

**Affiliations:** 1 Service de Neurochirurgie, Pôle des Neurosciences, Centre Hospitalier Universitaire de Rennes, 35000 Rennes, France; 2 Faculté de Médecine, Université de Rennes 1, 35000 Rennes, France; 3 Faculté de Médecine, Université de Genève, 1205 Geneve, Switzerland; 4 Laboratoire du Traitement de Signal, Unité Médicis, INSERM UMR 1099 LTSI, Université de Rennes 1, 35000 Rennes, France; 5 Department of Neurosurgery, Geneva University Hospitals, 1205 Geneva, Switzerland; 6 Besta NeuroSim Center, Fondazione IRCCS, Istituto Neurologico Carlo Basta, 20133 Milano, Italy

**Keywords:** meningioma, recurrence, overall survival, progression-free survival, retreatment

## Abstract

**Simple Summary:**

Intracranial meningiomas account for 30% to 40% of the primary lesions of the central nervous system. Surgery is the mainstay treatment whenever symptoms related to an intra-cranial meningioma are encountered. However, the management of recurrences after initial surgery, which are not uncommon, is still a matter of debate. Here, we present the alternatives described in the management of meningioma recurrence (radiotherapy, stereotaxic radiosurgery, protontherapy, and chemotherapy, among others). Their overall results are compared to surgery and future perspectives are presented.

**Abstract:**

Background: While meningiomas often recur over time, the natural history of repeated recurrences and their management are not well described. Should recurrence occur, repeat surgery and/or use of adjuvant therapeutic options may be necessary. Here, we summarize current practice when it comes to meningioma recurrence after initial surgical management. Methods: A total of N = 89 articles were screened. N = 41 articles met the inclusion criteria and N = 16 articles failed to assess management of meningioma recurrence. Finally, N = 24 articles were included in our review. Results: The articles were distributed as follows: studies on chemotherapy (N = 14), radiotherapy, protontherapy, and stereotaxic radiosurgery (N = 6), boron-neutron capture therapy (N = 2) and surgery (N = 3). No study seems to provide serious alternatives to surgery in terms of progression-free and overall survival. Recurrence can occur long after the initial surgery and also affects WHO grade 1 meningiomas, even after initial gross total resection at first surgery, emphasizing the need for a long-term and comprehensive follow-up. Conclusions: Surgery still seems to be the state-of-the-art management when it comes to meningioma recurrence, since none of the non-surgical alternatives show promising results in terms of progression-free and overall survival.

## 1. Introduction

Intracranial meningiomas are amongst the most common intracranial tumors and are regularly encountered in neurosurgical practice. Their initial management is often straightforward and treatment is typically advocated when symptoms are present or if growth is observed on serial imaging [1]. In this case, maximal safe tumor resection, as well as complete removal of the dural tail, is advocated [1,2]. Depending on size, location, and anatomical relationship with the surrounding structures, achieving a gross total resection (GTR) can be challenging [2,3,4,5,6,7,8,9,10] and may not even be worth the risk of surgery-related morbidity and functional impairment [5,11]. This is particularly true for skull base meningiomas (SBMs), where a radical excision may represent a challenge and sometimes even be detrimental, especially when cranial nerve and vascular structures are involved [5,7,8,12,13,14,15]. In this perspective, meningiomas might be viewed as a chronic condition rather than a tumor to be eradicated at all costs. 

The classic description of the extent of resection (EOR) of meningiomas relies on the surgeon’s intraoperative assessment of whether the tumor has been biopsied, subtotally removed, completely removed, or completely removed with additional resection of dural and bone invasion, as described by Simpson in 1957 [16]. However, the Simpson grading represents the surgeon’s subjective evaluation [17], it is less sensitive for SBMs [2,18,19], and the risk of residual tumor in the resection margins exists even after Simpson grade 1 surgery [20]. Yet, the EOR has been the most privileged prognostic factor of progression-free survival (PFS) for years [11,19,21,22,23,24,25,26], and it is now widely accepted that a complete resection yields superior disease control with a lower risk of recurrence in low-grade meningiomas and increased overall survival (OS) in high-grade meningiomas [2,10,16,17,27].

Meningiomas often recur over time, regardless of the initial EOR [10,25,26], and repeat surgery and/or use of adjuvant therapeutic options may be necessary. While there is abundant literature on the initial management of meningiomas, the natural history of repeated recurrences and their management is less well described [10,11,13,28,29,30]. Our aim was to summarize current practice when it comes to meningioma recurrence after initial surgical management.

## 2. Methods

A systematic review was conducted according to the Preferred Reporting Items for Systematic Reviews and Meta-Analyses (PRISMA) 2020 guidelines [31]. No registration was required for this study. 

On 6 June 2022, a search of literature in Embase, Cochrane Library, PubMed, Google Scholar, and Web of Science was performed. We included literature from January 2012 to May 2022. The following Medical Subject Heading (MeSH) terms were used: “meningioma” AND “recurrence” AND “treatment” OR “management” OR “adjuvant” OR “therapy” OR “surgery” OR “retreatment” OR “re-operation” OR “radiotherapy” OR “radiosurgery” OR “proton-therapy” OR “gamma-knife”, resulting in a list of N = 307 articles. 

The inclusion criteria were: (1) peer-reviewed research articles, retrospective or prospective in adult patients diagnosed with recurrent meningioma; (2) histologically confirmed meningioma; (3) number of cases > 5 patients; (4) studies assessing either chemotherapy, radiotherapy (RT), proton-therapy (PT), brachytherapy (BT), stereotaxic radiosurgery (SRS), boron-neutron capture therapy (BNCT) or surgery; and (5) studies written in English, French, German, or Italian language.

Exclusion criteria were: (1) tumors other than meningiomas; (2) publications other than original reports and redundant data of a single dataset; (3) editorials, technical notes, letters, review articles; and (4) studies assessing > 1 adjuvant therapy modality on meningioma recurrences. 

The titles and abstracts of all the articles were screened independently by the authors and all the relevant full-text copies were acquired. The following data items were considered: (1) study characteristics (author, year, sample size, study design); (2) intervention (surgery, RT, SRS, chemotherapy, other); (3) outcome measures (PFS and OS); and (4) duration of follow-up (FU) (Table 1). 

A PICO question (P: Patient/Problem, I: Intervention, C: Comparison, O: Outcome) was formulated to lead the selection process: the population was defined as adult patients with intracranial meningiomas, the intervention was any type of procedure performed, and the outcomes were PFS and OS.

Finally, the Appraisal of Guidelines for Research and Evaluation (AGREE) Reporting Checklist was performed to reach the highest possible quality of manuscript [54]. 

## 3. Results

### 3.1. Articles Included

After careful review of abstracts, a total of N = 89 articles were included and screened. N = 41 articles met the inclusion criteria and N = 16 articles failed to assess management of meningioma recurrence. Finally, N = 24 articles were included in our review [22,29,32,33,34,35,36,37,38,39,40,41,42,43,44,45,46,47,49,50,51,52,53,55,56,57] (Flowchart 1). The articles were distributed as follows: studies on chemotherapy (N = 14); RT, PT, and SRS (N = 6); BNCT (N = 2); and surgery (N = 3). Date of publication, design, primary and secondary endpoints of the studies are summarized in Table 1.

### 3.2. Chemotherapy

A total of N = 14 studies assessing the efficiency of chemotherapeutic agents were retrieved and distributed as follows: >1 drugs assessed (N = 1); Protein-kinase inhibitors (PKI; N = 3); Sunitinib Malate (N = 2); Hydroxyurea (HU; N = 5); Somatostatin analogues (SSA; N = 2); other drugs (N = 2). Studies assessing chemotherapy in recurring meningiomas are summarized in Table 1.

#### 3.2.1. Multiple Chemotherapeutic Agents

The radiologic effect of various chemotherapeutic agents on recurring meningiomas has been studied by Furtner et al. [41] in the perspective of growth rate, tumor volume, diameter, and size of tumor-associated edema. The authors evaluated and compared the role of bevacizumab, somatostatin analogues, tyrosine kinase inhibitors (TKI), and other cytotoxic agents in N = 34 patients. Overall, growth rate decreased during therapy by 51% and 14% for diameter and volume, respectively. In this study, bevacizumab appeared to be the most powerful agent showing a reduction of 80% in diameter and 59% in volume and being the only drug acting on peritumoral edema.

##### Protein-Kinase Inhibitors 

Three distinct phase II trials assessed the role of protein-kinase inhibitors (PKI) as adjuvant drugs in recurrent, surgery- and radiation-refractory WHO grade 1–3 meningiomas [36,42,44]. PKI were administered either as stand-alone therapy [36], or combined with somatostatin analogues [44] or anti-VEGF [42]. 

In a study from Raizer et al. [36], the authors used TKI PTK787 in N = 25 patients, of whom N = 11 patients had prior systemic chemotherapy. In this study, the majority of patients had WHO grade 2 and 3 meningiomas (N = 14 and N = 8, respectively). The results show that at best, a stable disease is observed, with PFS at six months (PFS-6) of 64.3% in WHO grade 2 patients and median PFS and OS of 6.5 months and 26 months, respectively. In WHO grade 3 patients, PFS-6 was 37.5% and median PFS and OS were 3.6 months and 23 months, respectively. 

Graillon et al. [44] investigated the efficacy of the combination of everolimus and octreotide in N = 20 patients with a majority of WHO grade 2–3 tumors (N = 18), showing a decrease in growth rate at 3 months in almost 80% of the tumors. More specifically, the overall growth rate fell from 16.6% before inclusion to 0.02% at 3 months, and 0.48% at 6 months post-inclusion [44]. 

The combination of everolimus with bevacizumab was tested by Shih et al. [42] in N = 17 patients suffering from WHO grade 1–3 recurrent meningiomas (29%, 41%, and 24% of WHO grade 1, 2 and 3 respectively). The overall median PFS of the cohort was 22 months (95% CI 4.5–26.8) and was greater for patients with WHO grade 2 and 3 compared to grade I tumors (22.0 months vs. 17.5 months). 

##### Sunitinib Malate 

Sunitinib malate is a TKI targeting specifically vascular endothelial growth factor receptors (VEGFR), platelet-derived growth-factor receptor (PDGFR), and CD117. 

Kaley et al. [38] showed the link between VEGFR expression and PFS using sunitinib malate in a cohort of N = 36 patients with surgery and radiation-refractory recurrent WHO grades 2–3 meningiomas (Phase II trial). Patients with VEGFR-negative tumors had a median PFS of 1.4 months, while patients with VEGFR-positive meningiomas had a median PFS of 6.4 months (*p* < 0.05), The overall PFS-6 of the cohort was 42%, while the median PFS was 5.2 months (95% CI: 2.8–8.3). The median OS was 24.6 months (95% CI: 16.5–38.4). 

Later, Cardona et al. [43] compared the effectiveness of sunitinib versus octreotide and everolimus in a cohort of N = 31 patients with recurrent WHO grade 2 and 3 meningiomas. N = 14 patients received combination therapy with octreotide/everolimus and N = 11 received sunitinib alone, while N = 6 patients received other therapy. The PFS in the octreotide/everolimus group was 12.1 months (95% CI 9.2–21.1) versus 9.1 months (95% CI 6.8–16.8) in the cohort treated with sunitinib (*p* = 0.43), showing how the expression of VEGFR and PDGFR could be linked to better outcomes [43]. 

##### Hydroxyurea 

HU was assessed in N = 5 studies [32,34,35,39,55] of which N = 2 were phase II trials [35,55]. HU was assessed either alone [32,34] or in combination with other anti-neoplastic drugs [35,39,55]. 

Mason et al. [32] focused on recurrent, unresectable WHO grade 1–3 meningiomas involving dural venous sinus in N = 20 patients. In patients with WHO grade 1 lesions (N = 16), the PFS-12 was 93%, while tumor progression was observed in all the remaining patients. 

Kim et al. [34] treated N = 13 patients showing recurrent WHO grade 1–2 meningiomas with HU. N = 10 patients showed disease stability with PFS ranging from 8–128 months (median 77 months). 

Reardon et al. [35] combined HU and imatinib in N = 21 patients; PFS-6 was 61.9%, 87.5%, and 46.2% in WHO grade 1, 2, and 3, respectively, while Mazza et al. [55] assessed the same association in N = 15 patients and compared it to a protocol with HU alone. The trial closed prematurely due to slow enrollment rate but the association of HU with imatinib versus HU alone was rather in favor of HU, since the two groups showed significantly different median PFS at 9 months post-inclusion (4.0 versus 19.5 months, respectively).

Karsy et al. [39] combined HU to verapamil to treat WHO grade 1–2 meningiomas in N = 7 patients [39], with no radiographic response during FU. The median PFS was 8 months, while PFS-6 was 85%. 

##### Somatostatin Analogues

SSAs were used in N = 2 phase II trials [37,40,48]. 

Simò et al. [37] used octreotide in N = 9 patients with WHO grade 2–3. The median time-to-progression was 4.2 months (1.0–9.4) and all patients had tumor progression 10 months after inclusion. Norden et al. [40] assessed the efficacy of pasireotide in N = 34 patients with recurring WHO grade 1–3, but failed to find any radiographic response during FU.

##### Other Drugs

Chamberlain et al. [33] assessed the role of irinotecan, a topoisomerase I inhibitor, in recurring, radio-resistant WHO grade 1 meningiomas in a phase II trial involving N = 16 patients, using the PFS-6 as primary outcome. Since no patient showed PFS-6, the study was stopped prematurely. 

Recently, Belanger et al. [45] retrospectively assessed PFS and OS in N = 11 patients who underwent adjuvant therapy using temozolomide (TMZ) in addition to postoperative RT, with a median follow-up of 41.5 months. While N = 3 patients died during FU, the N = 2 patients with WHO grade 3 meningiomas showed recurrence less than three months after initiation of adjuvant therapy. Three-year OS and PFS for WHO grade 2 meningiomas were both 88%, with a 3-year median OS and PFS of 83% and 75.8%, respectively [45]. 

### 3.3. Radiotherapy, Brachytherapy, and Stereotaxic Radiosurgery

A total of N = 5 studies assessing the efficiency of RT, PT, and BT were retrieved.

Bartolomei et al. [46] used (90)Y-DOTATOC in N = 29 patients with recurring WHO grade 1–3 meningiomas with strong expression of somatostatin receptors. Magnetic resonance imaging (MRI) showed stabilization in 66%, while 34% had disease progression. The median PFS was 61 and 13 months in low- and high-grade meningiomas, respectively. A similar protocol was reported by Gerster-Gillieron et al. [48], an overall PFS exceeding 24 months in patients with WHO grade 1–3 tumors.

Champeaux-Depond et al. [49] reviewed cases of patients who underwent proton-therapy following meningioma recurrence or progression after initial surgery: the authors included data from N = 193 patients, with a median FU of 4.4 years (IQR 4.85–4.71). The five-year PFS were 71.5% (95% CI 64.4–79.4), 55.6% (95% CI 32.5–95), and 35.6% (95% CI 12.8–98.9) for WHO grade 1, 2, and 3 meningiomas, respectively. The 5-years OS rates were 93% (95% CI 88.7–97.4), 76.4% (95% CI 51.4–100), and 44.4% (95% CI 16.7–100) for WHO grade 1, 2, and 3 meningiomas, respectively [49].

Wojcienszynski et al. [47] reviewed data from N = 19 patients with meningioma initially treated with SRS or fractionated stereotactic RT, of which N = 11 presented recurrence requiring re-irradiation. The overall median time-to-second progression was 10 months while PFS at one year was reduced in patients with higher grade meningiomas compared to patients with benign histology. The median PFS for patients with WHO grade 2–3 was 8 months while it was not reached for patients with benign histology. Altogether, these results indicate that reirradiation is not an option in higher grade meningiomas. 

Gallagher et al. [22] retrospectively analyzed their series of N = 145 patients with WHO grade 1 meningiomas undergoing surgical resection with a median FU of 60 months. N = 10 cases had recurrence/progression at a median period of 42 months. Of these, four remained under active surveillance, three received SRS, and three were treated with RT. 

#### Boron Neutron Capture Therapy (BNCT)

N = 2 studies assessed the efficacy of BNCT in recurring meningiomas. Takai et al. [51] used BNCT in N = 44 patients with high grade meningiomas and reported their results in a retrospective, single-center study. The authors analyzed the OS after initial diagnosis and after BNCT as well as PFS after BNCT. The median FU after BNCT was 26 months (6.4–103) and median OS after BNCT was 29.6 months (95% CI 16.1–40.4), while the median OS after initial diagnosis was 98.4 months (95% CI: 68.7–169.4). N = 35 tumors showed shrinkage during the observation period. The median PFS post-BNCT was 13.7 months (95% CI 8.3–28.6) and local recurrence was observed in 22% of cases. 

Takeuchi et al. [50] reported their results using BNCT in patients with high-grade recurring meningiomas. In patients with SBM, OS were 24.6 and 67.5 months after BCNT and diagnosis, respectively. 

### 3.4. Surgery

N = 3 studies assessed the efficacy of surgery in recurring meningiomas. In a mixed retro- and prospective study, Lemée et al. [29] reported a cohort of N = 1469 patients with surgically managed meningiomas, in which N = 114 recurrences occurred. The risk of having a surgical re-treatment was 1% per patient-year of follow-up (total 11,414 patient-years of follow-up), with a decreasing time-to-retreatment after repeated surgeries for recurrences (from 4.3 ± 5 years after initial surgery to 2.4 ± 2.9 years after third surgery) [29]. 

Richardson et al. [53] investigated the outcomes of N = 56 patients who had re-operation for meningioma recurrence. The median time to re-operation after initial surgery was 35 months (95% CI 28.9–41.1), while it was 68 months (95% CI 49.1–86.9) after re-operation. The median OS was 312.0 months (95% CI 257.8–366.2).

In a retrospective review, Magill et al. [52] analyzed data from patients with recurrent SBM, with a median FU of 8.5 years. A total of N = 78 patients underwent re-operation, N = 17 had a second-re-operation, and N = 3 had a third recurrence requiring surgery. The median time to-reoperation was 4.4 years after initial surgery, and 4.1 years from the second to the third surgery. The 1-, 2-, 5-, and 10-year OS rates after the first reoperation were 94%, 92%, 88%, and 76%, respectively. The median survival after the first reoperation was 17 years.

## 4. Discussion

### 4.1. Recurrence and Long-Term Follow-Up

Our aim was to summarize current practices in the management of intra-cranial meningioma recurrences after initial surgery, from the perspective of chemotherapy and radiotherapy (and other assimilated therapies) as well as surgery. Although the results are heterogeneous and no study seems to provide serious alternatives to surgery in terms of PFS and OS, the results allow us to grasp two important aspects: (1) recurrence can occur long after the initial surgery, which implies that the FU must be extended, and (2) recurrence certainly affects WHO grade 2 and 3 meningiomas (sometimes early or ultra-early), but also benign meningiomas, even after achieving GTR at first surgery, emphasizing again the need for a long-term and comprehensive follow-up [10]. 

The need for a long-term follow-up is well illustrated by the study from Pettersson-Segerlind et al. [10], which analyzed the 25-years recurrence rate of a cohort of N = 51 patients operated on for parasagittal meningiomas between 1975–1979, showing a recurrence rate as high as 47%. 

### 4.2. Lack of Data and Heterogeneity

When considering the frequent occurrence of intracranial meningiomas, we were surprised by the paucity of studies focusing on non-surgical management. Aside from being rare, the studies often involve only few patients and combine drugs and pathologies; i.e., do we include apples and oranges when WHO grade 1 and 2/3 are studied in the same protocol? Lastly, some studies failed to show any positive results [33,39,40] or were even stopped prematurely due to slow inclusion [35]. Altogether, data retrieved are rather weak and might be questionable in the perspective of their applicability to larger cohorts.

For example, while Furtner et al. [41] describe a very encouraging radiographic response when recurrent meningiomas were treated with various chemotherapeutic agents and the use of everolimus in combination with octreotide or bevacizumab may be useful in WHO grade 2 and 3 meningiomas [42,44], no drugs among PKI, sunitinib malate, HU, SSA, irinotecan, or temozolomide seem to be really promising at this point. 

A similar observation can be made from results of cohorts treated with RT, BT, PT, and SRS as stand-alone therapies of recurring meningiomas. Furthermore, re-irradiation of initially irradiated meningiomas does not seem to carry benefits in terms of PFS in WHO grade 2–3, which are precisely the lesions for which irradiation would be most beneficial [47]. This also raises the question of the time-interval between irradiating treatments, their modalities (SRS, RT, other), and the dose that should be used. Results of trials such as the ROAM study (Radiation versus Observation following surgical resection in atypical meningiomas) are expected to shed light on these issues [58].

Lastly, only a single article assessed the role of PT in recurring meningiomas. This is surprising since PT has been shown to be effective and safe in the treatment of low-grade meningiomas, especially in cases of SBMs, complex or radiation-induced meningiomas [59,60,61,62,63,64,65,66,67].

### 4.3. Bias towards Surgery 

Lemée et al. [29] and Richardson et al. [53] have shown that PFS after re-surgery is far longer than after non-surgical adjuvant therapies. Certainly, this may be regarded as an argument in favor of surgery for recurring meningiomas, as it is shown by the cohort of recurring SBMs described by Magill et al. [52]. However, populations and tumors studied may differ in terms of patients baseline demographics, tumor locations, size, and grades. Altogether, this may bias our perception of surgery being the best answer for recurring meningiomas.

This may result in a situation where a lack of efficient non-surgical adjuvant therapies leads to a quasi-dichotomous situation where either “nothing” (i.e., observation) or re-operation occur. When it comes to dealing with higher grade meningiomas, the management of recurrence is even more complex, especially when GTR cannot (or must not) be achieved. This leads to a vision that maybe misses the current general consensus where a patient-tailored management, emphasizing functional preservation and quality of life, based on a case-by-case discussion in accordance with the overall guidelines of the European Association of Neuro-Oncology [1] is increasingly advocated. In our opinion, this vision should be supported by clear-cut guidelines in cases of recurrence or progression. 

Lastly, trials assessing alternatives to re-surgery in recurring meningiomas are regularly brought to the field, illustrating a constant effort to improve patient care and overall outcomes. Still, data are scarce and heterogeneously reported, and results demonstrate how difficult it is to find the one-size-fits-all solution. As an example, Klinger et al. [68] reported data from N = 19 patients developing recurrence of atypical meningiomas initially treated by surgery, using repeat surgery and radiation therapy (either gamma-knife, cyberknife, or intensity-modulated radiation therapy), were treated with radiation therapy alone or surgery alone. Although the survival rate at last FU was 95.3%, it is difficult to determine whether it’s related to surgery or radiotherapy [68].

### 4.4. Risk Factors of Recurrence

Amongst predictors of recurrence, the WHO grade and location are the strongest [3,8,29], while clinical and radiological parameters are also to be considered [69]. The role of genomics and genetic profiling is being increasingly discussed [70,71,72,73]. Besides, PFS is directly related to the EOR in any WHO grade. Simpson grades 1, 2, and 3 are considered GTR by the European Association of Neuro-Oncology (EANO) [1]; while Simpson grade 3 is compatible with complete removal, the risk of residual tumor in margins, surrounding inflammatory tissues, meninges and bone is relatively high, as is the risk of recurrence [17,24,74]. 

Predictors of incomplete resection have been described elsewhere [11] and are predominantly: (1) symptomatic presentation; (2) skull-base location, and (3) bone invasion. For example, Da Broi et al. [28] showed that in subtotally resected WHO grade 1 SBMs, re-treatment rate may rise up to 16%, 27%, 34%, and 38% at 1, 3, 5, and 10-years follow-up, illustrating how frequently recurrences are encountered after surgery [28]. Besides, Kuranari et al. [75] assessed the predictors of shorter PFS in SBMS after initial surgical management: the 2-, 5-, and 10-year PFS rates were 68%, 53%, and 23%, respectively. The authors showed that higher WHO grades, multiple lesions, and tumor size were associated with shorter PFS, while postoperative radiotherapy improved PFS in patients with WHO grade 2 lesions after initial subtotal resection. Altogether, these data suggest that when confronted to locations (or any other predictor) associated with an increased risk for recurrence, it may be of interest to discuss upfront SRS/SRT or systematic adjuvant therapy. 

On the other hand, Al-Mefty et al. [76] showed radiation-induced meningiomas are more aggressive lesions with extremely elevated rates of recurrences, along with higher histopathological grade and complex cytogenetic aberrations. This goes along with the findings of Couldwell et al. [77], showing how skull-base meningioma can grow aggressively after radiosurgery, even in the long-term. In this case, observation is an option in the first instance, but only at the price of long-term, comprehensive, and expert-supported policy. 

### 4.5. Detecting Recurrences

Previously, Lemée et al. [29] reported a decreasing time-to-retreatment in repeated surgeries for recurrences (from 4.3 ± 5 years after initial surgery to 2.4 ± 2.9 years after third surgery), supporting further the need for accurate and comprehensive follow-up protocols. 

Most studies use variable follow-up policies, as EANO recommendations are based on consensus opinion of experts rather than scientific evidence, resulting in varying intervals between FU visits, according to tumor and patient’s characteristics. 

As an alternative to the Simpson grading scale, the postoperative MRI allows the objective and early assessment of the EOR [1] during FU; still, meningioma cells have the potential to invade adjacent mesenchymal tissues at a microscopic level [78] and tumor remnants can be easily be overlooked, even with high-field MRI [79]. Recently, Boto et al. [80] challenged the pertinence of contrast-enhanced MRI in the follow-up of patients with untreated intracranial meningiomas, showing that the use of 3D-T2 weighted images could be as accurate as T1 3D-gadolinium sequences. Given concerns relating to repeat administration of gadolinium-based contrast may lead to depositions in the dentate nucleus and the globus pallidus [81], non-contrast MR imaging should probably be favored when possible. 

Recent studies show that imaging techniques other than MRI may be superior to detect recurrence during FU. Ueberschaer et al. [82] compared the use postoperative MRI and 68Ga-DOTATATE/PET-CT within 6 months after surgery: PET-CT showed uptake in 15/37 patients with GTR (defined as Simpson grades 1 and 2), suggesting unexpected tumor remnants (41% false negative) and MRI was false negative in 7 of these 15 cases (19% false negative) (*p* = 0.037), showing how PET-CT improves detection rates compared to MRI. These results were corroborated by Gay et al. [83]. 

The Copenhagen Grading (postoperative PET-MRI imaging with 68Ga-DOTATOC) was proposed to provide a more sensitive and specific imaging than MRI following surgery of meningiomas [84]. Since almost all meningiomas overexpress SSTR-2, identification of residual tumor even in the mm range is feasible by combining the radiotracer SSTR-2 ligand Ga-68 DOTATOC and PET-MRI performed on a high-resolution research tomograph [85,86]. 

### 4.6. The Role of the Methylome as Predictor of Tumor Recurrence and Prognosis

Molecular biology and genomics represent an opportunity for the development of future prognostic tools that could be implemented in the daily practice and treatment of meningiomas, as is already the case in the management of gliomas. The methylation profiling has been shown to better define subgroups of meningiomas [71,87], when it comes to elaborate the risk of recurrence and the overall prognostics of a meningioma. This should also be considered when confronted with recurrences. Hence, the role of the DNA-methylation-based classification of meningiomas should be considered not only when confronted with a meningioma after initial recurrence, but also whenever recurrence is observed. Trials comparing observation and early adjuvant treatment after first surgery (and even re-surgery) should be elaborated with the perspective DNA-methylation analysis. 

## 5. Strengths and Limitations

Due to the heterogeneity of the data, the uniform reporting of outcomes in terms of OS and PFS was not possible/relevant. The literature search was obviously also limited by a large quantity of articles reporting data from very small cohorts, limited FU, and mixed management of recurrences. Still, the major publications in the field are listed and discussed, highlighting the lack of consensus and the variability of treatment.

## Figures and Tables

**Flowchart 1:**
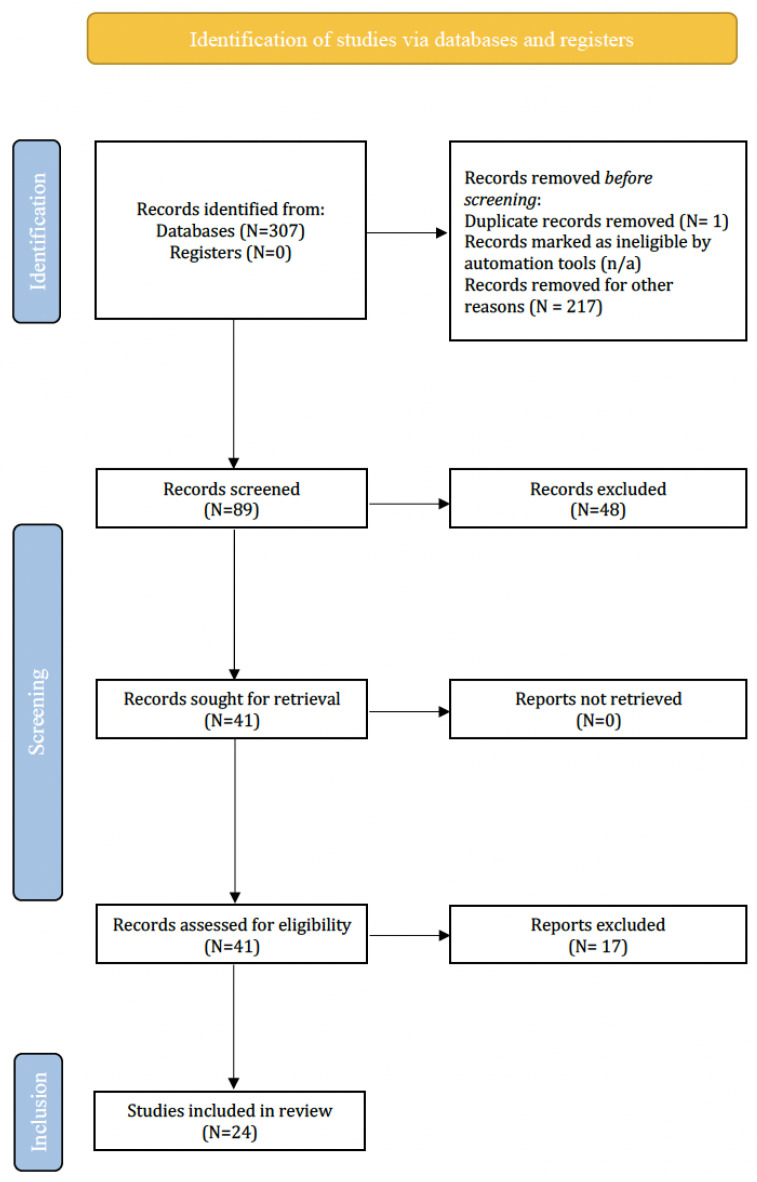
Preferred Reporting Items for Systematic Reviews and Meta-Analyses (PRISMA) flow diagram of the literature review.

**Table 1:** List of articles encompassed in the review, including their design as well as the primary and secondary outcome measures. N: Number of patients; P: Prospective; R: Retrospective; PFS: Progression-free survival; OS: Overall survival.

	Author	Year	N	Design	Primary Endpoint	Secondary Endpoint
	**Chemotherapy**					
1	Mason et al. [32]	2002	20	P	Radiographic assessment	12-months-PFS; safety
2	Chamberlain et al. [33]	2006	16	P	6 months-PFS	OS, radiographic response, safety
3	Kim et al. [34].	2012	13	P	PFS	-
4	Reardon et al. [35]	2012	21	P	6 months-PFS	Radiographic response rate, OS and safety
5	Raizer et al. [36]	2014	25	P	6 months-PFS	OS, radiographic response
6	Simò et al. [37]	2014	9	P	Radiographic assessment	OS, PFS
7	Kaley et al. [38]	2015	36	P	6 months-PFS	Radiographic response rate, safety, PFS, OS
8	Karsy et al. [39]	2015	7	P	Radiographic assessment	PFS, safety
9	Norden et al. [40]	2015	34	P	6 months-PFS	Radiographic response rate, OS and safety
10	Furtner et al. [41]	2016	34	R	Radiographic assessment	-
11	Shih et al. [42]	2016	18	P	PFS	Radiographic response rate, OS and safety
12	Cardona et al. [43]	2019	31	P	PFS	OS, toxicity
13	Graillon et al. [44]	2020	20	P	6 months-PFS	PFS; OS; 3D Tumor growth; SSTR2A expression; NF2 mutation identification; safety
14	Belanger et al. [45]	2022	11	P	PFS	OS
	**Radiotherapy and protontherapy**					
15	Bartolomei et al. [46]	2009	29	P	PFS	OS
16	Wojcienszynski et al. [47]	2012	19	R	PFS	-
17	Gerster-Gilliéron et al. [48]	2015	15	P	PFS	Toxicity
18	Gallagher et al. [22]	2016	145	R	PFS	-
19	Champeaux-Depond et al. [49]	2021	193	R	PFS	OS
	**Boron neutron capture therapy**					
20	Takeuchi et al. [50]	2018	31	R	Radiographic assessment	OS
21	Takai et al. [51]	2022	44	R	Radiographic assessment	OS, PFS
	**Surgery**					
22	Magill et al. [52]	2018	78	Retrospective	PFS	OS
23	Lemée et al. [29]	2020	1469	Retrospective	PFS	OS
24	Richardson et al. [53]	2021	56	Retrospective	WHO performance	PFS, OS

## References

[1] Goldbrunner R., Stavrinou P., Jenkinson M.D., Sahm F., Mawrin C., Weber D.C., Preusser M., Minniti G., Lund-Johansen M., LaFrance F. (2021). EANO guideline on the diagnosis and management of meningiomas. Neuro-Oncology.

[2] Hasseleid B.F., Meling T.R., Ronning P., Scheie D., Helseth E. (2012). Surgery for convexity meningioma: Simpson Grade I resection as the goal: Clinical article. J. Neurosurg..

[3] Corniola M.V., Lemee J.M., Da Broi M., Joswig H., Schaller K., Helseth E., Meling T.R. (2019). Posterior fossa meningiomas: Perioperative predictors of extent of resection, overall survival and progression-free survival. Acta Neurochir..

[4] Corniola M.V., Lemee J.M., Schaller K., Meling T.R. (2019). Lateral sphenoid wing meningiomas without bone invasion-still skull base surgery?. Neurosurg. Rev..

[5] Giammattei L., di Russo P., Starnoni D., Passeri T., Bruneau M., Meling T.R., Berhouma M., Cossu G., Cornelius J.F., Paraskevopoulos D. (2021). Petroclival meningiomas: Update of current treatment and consensus by the EANS skull base section. Acta Neurochir..

[6] Giammattei L., Starnoni D., Cossu G., Bruneau M., Cavallo L.M., Cappabianca P., Meling T.R., Jouanneau E., Schaller K., Benes V. (2020). Surgical management of Tuberculum sellae Meningiomas: Myths, facts, and controversies. Acta Neurochir..

[7] Meling T.R., Da Broi M., Scheie D., Helseth E. (2019). Skull base versus non-skull base meningioma surgery in the elderly. Neurosurg. Rev..

[8] Meling T.R., Da Broi M., Scheie D., Helseth E. (2019). Meningiomas: Skull base versus non-skull base. Neurosurg. Rev..

[9] Starnoni D., Tuleasca C., Giammattei L., Cossu G., Bruneau M., Berhouma M., Cornelius J.F., Cavallo L., Froelich S., Jouanneau E. (2021). Surgical management of anterior clinoidal meningiomas: Consensus statement on behalf of the EANS skull base section. Acta Neurochir..

[10] Pettersson-Segerlind J., Orrego A., Lonn S., Mathiesen T. (2011). Long-term 25-year follow-up of surgically treated parasagittal meningiomas. World Neurosurg..

[11] Lemee J.M., Corniola M.V., Da Broi M., Joswig H., Scheie D., Schaller K., Helseth E., Meling T.R. (2019). Extent of Resection in Meningioma: Predictive Factors and Clinical Implications. Sci. Rep..

[12] Lemee J.M., Joswig H., Da Broi M., Corniola M.V., Scheie D., Schaller K., Helseth E., Meling T.R. (2020). WHO grade I meningiomas: Classification-tree for prognostic factors of survival. Neurosurg. Rev..

[13] Mathiesen T., Lindquist C., Kihlstrom L., Karlsson B. (1996). Recurrence of cranial base meningiomas. Neurosurgery.

[14] Nakao N., Ohkawa T., Miki J., Nishibayahsi H., Ogura M., Uematsu Y., Itakura T. (2011). Analysis of factors affecting the long-term functional outcome of patients with skull base meningioma. J. Clin. Neurosci..

[15] Seifert V. (2010). Clinical management of petroclival meningiomas and the eternal quest for preservation of quality of life: Personal experiences over a period of 20 years. Acta Neurochir..

[16] Simpson D. (1957). The recurrence of intracranial meningiomas after surgical treatment. J. Neurol. Neurosurg. Psychiatry.

[17] Schwartz T.H., McDermott M.W. (2020). The Simpson grade: Abandon the scale but preserve the message. J. Neurosurg..

[18] Meling T.R. (2012). Editorial: Response: Simpson grades. J. Neurosurg..

[19] Voss K.M., Spille D.C., Sauerland C., Suero Molina E., Brokinkel C., Paulus W., Stummer W., Holling M., Jeibmann A., Brokinkel B. (2017). The Simpson grading in meningioma surgery: Does the tumor location influence the prognostic value?. J. Neurooncol..

[20] Jaaskelainen J. (1986). Seemingly complete removal of histologically benign intracranial meningioma: Late recurrence rate and factors predicting recurrence in 657 patients. A multivariate analysis. Surg. Neurol..

[21] Rydzewski N.R., Lesniak M.S., Chandler J.P., Kalapurakal J.A., Pollom E., Tate M.C., Bloch O., Kruser T., Dalal P., Sachdev S. (2018). Gross total resection and adjuvant radiotherapy most significant predictors of improved survival in patients with atypical meningioma. Cancer.

[22] Gallagher M.J., Jenkinson M.D., Brodbelt A.R., Mills S.J., Chavredakis E. (2016). WHO grade 1 meningioma recurrence: Are location and Simpson grade still relevant?. Clin. Neurol. Neurosurg..

[23] Ehresman J.S., Garzon-Muvdi T., Rogers D., Lim M., Gallia G.L., Weingart J., Brem H., Bettegowda C., Chaichana K.L. (2019). Risk of Developing Postoperative Deficits Based on Tumor Location after Surgical Resection of an Intracranial Meningioma. J. Neurol. Surg. B Skull Base.

[24] Gousias K., Schramm J., Simon M. (2016). The Simpson grading revisited: Aggressive surgery and its place in modern meningioma management. J. Neurosurg..

[25] Mirimanoff R.O., Dosoretz D.E., Linggood R.M., Ojemann R.G., Martuza R.L. (1985). Meningioma: Analysis of recurrence and progression following neurosurgical resection. J. Neurosurg..

[26] Adegbite A.B., Khan M.I., Paine K.W., Tan L.K. (1983). The recurrence of intracranial meningiomas after surgical treatment. J. Neurosurg..

[27] Aizer A.A., Bi W.L., Kandola M.S., Lee E.Q., Nayak L., Rinne M.L., Norden A.D., Beroukhim R., Reardon D.A., Wen P.Y. (2015). Extent of resection and overall survival for patients with atypical and malignant meningioma. Cancer.

[28] Da Broi M., Borrelli P., Meling T.R. (2021). Predictors of Survival in Subtotally Resected WHO Grade I Skull Base Meningiomas. Cancers.

[29] Lemee J.M., Corniola M.V., Meling T.R. (2020). Benefits of re-do surgery for recurrent intracranial meningiomas. Sci. Rep..

[30] Kent C.L., Mowery Y.M., Babatunde O., Wright A.O., Barak I., McSherry F., Herndon J.E., Friedman A.H., Zomorodi A., Peters K. (2022). Long-Term Outcomes for Patients with Atypical or Malignant Meningiomas Treated with or Without Radiation Therapy: A 25-Year Retrospective Analysis of a Single-Institution Experience. Adv. Radiat. Oncol..

[31] Page M.J., McKenzie J.E., Bossuyt P.M., Boutron I., Hoffmann T.C., Mulrow C.D., Shamseer L., Tetzlaff J.M., Akl E.A., Brennan S.E. (2021). The PRISMA 2020 statement: An updated guideline for reporting systematic reviews. BMJ.

[32] Mason W.P., Gentili F., Macdonald D.R., Hariharan S., Cruz C.R., Abrey L.E. (2002). Stabilization of disease progression by hydroxyurea in patients with recurrent or unresectable meningioma. J. Neurosurg..

[33] Chamberlain M.C., Tsao-Wei D.D., Groshen S. (2006). Salvage chemotherapy with CPT-11 for recurrent meningioma. J. Neurooncol..

[34] Kim M.S., Yu D.W., Jung Y.J., Kim S.W., Chang C.H., Kim O.L. (2012). Long-term follow-up result of hydroxyurea chemotherapy for recurrent meningiomas. J. Korean Neurosurg. Soc..

[35] Reardon D.A., Norden A.D., Desjardins A., Vredenburgh J.J., Herndon J.E., Coan A., Sampson J.H., Gururangan S., Peters K.B., McLendon R.E. (2012). Phase II study of Gleevec(R) plus hydroxyurea (HU) in adults with progressive or recurrent meningioma. J. Neurooncol..

[36] Raizer J.J., Grimm S.A., Rademaker A., Chandler J.P., Muro K., Helenowski I., Rice L., McCarthy K., Johnston S.K., Mrugala M.M. (2014). A phase II trial of PTK787/ZK 222584 in recurrent or progressive radiation and surgery refractory meningiomas. J. Neurooncol..

[37] Simo M., Argyriou A.A., Macia M., Plans G., Majos C., Vidal N., Gil M., Bruna J. (2014). Recurrent high-grade meningioma: A phase II trial with somatostatin analogue therapy. Cancer Chemother. Pharm..

[38] Kaley T.J., Wen P., Schiff D., Ligon K., Haidar S., Karimi S., Lassman A.B., Nolan C.P., DeAngelis L.M., Gavrilovic I. (2015). Phase II trial of sunitinib for recurrent and progressive atypical and anaplastic meningioma. Neuro-Oncol..

[39] Karsy M., Hoang N., Barth T., Burt L., Dunson W., Gillespie D.L., Jensen R.L. (2016). Combined Hydroxyurea and Verapamil in the Clinical Treatment of Refractory Meningioma: Human and Orthotopic Xenograft Studies. World Neurosurg..

[40] Norden A.D., Ligon K.L., Hammond S.N., Muzikansky A., Reardon D.A., Kaley T.J., Batchelor T.T., Plotkin S.R., Raizer J.J., Wong E.T. (2015). Phase II study of monthly pasireotide LAR (SOM230C) for recurrent or progressive meningioma. Neurology.

[41] Furtner J., Schopf V., Seystahl K., Le Rhun E., Ruda R., Roelcke U., Koeppen S., Berghoff A.S., Marosi C., Clement P. (2016). Kinetics of tumor size and peritumoral brain edema before, during, and after systemic therapy in recurrent WHO grade II or III meningioma. Neuro-Oncol..

[42] Shih K.C., Chowdhary S., Rosenblatt P., Weir A.B., Shepard G.C., Williams J.T., Shastry M., Burris H.A., Hainsworth J.D. (2016). A phase II trial of bevacizumab and everolimus as treatment for patients with refractory, progressive intracranial meningioma. J. Neurooncol..

[43] Cardona A.F., Ruiz-Patino A., Zatarain-Barron Z.L., Hakim F., Jimenez E., Mejia J.A., Ramon J.F., Useche N., Bermudez S., Pineda D. (2019). Systemic management of malignant meningiomas: A comparative survival and molecular marker analysis between Octreotide in combination with Everolimus and Sunitinib. PLoS ONE.

[44] Graillon T., Sanson M., Campello C., Idbaih A., Peyre M., Peyriere H., Basset N., Autran D., Roche C., Kalamarides M. (2020). Everolimus and Octreotide for Patients with Recurrent Meningioma: Results from the Phase II CEVOREM Trial. Clin. Cancer Res..

[45] Belanger K., Ung T.H., Damek D., Lillehei K.O., Ormond D.R. (2022). Concomitant Temozolomide plus radiotherapy for high-grade and recurrent meningioma: A retrospective chart review. BMC Cancer.

[46] Bartolomei M., Bodei L., De Cicco C., Grana C.M., Cremonesi M., Botteri E., Baio S.M., Arico D., Sansovini M., Paganelli G. (2009). Peptide receptor radionuclide therapy with (90)Y-DOTATOC in recurrent meningioma. Eur. J. Nucl. Med. Mol. Imaging.

[47] Wojcieszynski A.P., Ohri N., Andrews D.W., Evans J.J., Dicker A.P., Werner-Wasik M. (2012). Reirradiation of recurrent meningioma. J. Clin. Neurosci..

[48] Gerster-Gillieron K., Forrer F., Maecke H., Mueller-Brand J., Merlo A., Cordier D. (2015). 90Y-DOTATOC as a Therapeutic Option for Complex Recurrent or Progressive Meningiomas. J. Nucl. Med..

[49] Champeaux-Depond C., Weller J. (2021). Outcome after Protontherapy for Progression or Recurrence of Surgically Treated Meningioma. Brain Tumor Res. Treat..

[50] Takeuchi K., Kawabata S., Hiramatsu R., Matsushita Y., Tanaka H., Sakurai Y., Suzuki M., Ono K., Miyatake S.I., Kuroiwa T. (2018). Boron Neutron Capture Therapy for High-Grade Skull-Base Meningioma. J. Neurol. Surg. B Skull Base.

[51] Takai S., Wanibuchi M., Kawabata S., Takeuchi K., Sakurai Y., Suzuki M., Ono K., Miyatake S.I. (2022). Reactor-based boron neutron capture therapy for 44 cases of recurrent and refractory high-grade meningiomas with long-term follow-up. Neuro-Oncol..

[52] Magill S.T., Lee D.S., Yen A.J., Lucas C.G., Raleigh D.R., Aghi M.K., Theodosopoulos P.V., McDermott M.W. (2018). Surgical outcomes after reoperation for recurrent skull base meningiomas. J. Neurosurg..

[53] Richardson G.E., Gillespie C.S., Mustafa M.A., Taweel B.A., Bakhsh A., Kumar S., Keshwara S.M., Ali T., John B., Brodbelt A.R. (2021). Clinical Outcomes Following Re-Operations for Intracranial Meningioma. Cancers.

[54] Brouwers M.C., Kerkvliet K., Spithoff K., Consortium A.N.S. (2016). The AGREE Reporting Checklist: A tool to improve reporting of clinical practice guidelines. BMJ.

[55] Mazza E., Brandes A., Zanon S., Eoli M., Lombardi G., Faedi M., Franceschi E., Reni M. (2016). Hydroxyurea with or without imatinib in the treatment of recurrent or progressive meningiomas: A randomized phase II trial by Gruppo Italiano Cooperativo di Neuro-Oncologia (GICNO). Cancer Chemother. Pharm..

[56] Poulen G., Vignes J.R., Le Corre M., Loiseau H., Bauchet L. (2020). WHO grade II meningioma: Epidemiology, survival and contribution of postoperative radiotherapy in a multicenter cohort of 88 patients. Neurochirurgie.

[57] Koch M.J., Agarwalla P.K., Royce T.J., Shih H.A., Oh K., Niemierko A., Mauceri T.C., Curry W.T., Barker F.G., Loeffler J.S. (2019). Brachytherapy as an Adjuvant for Recurrent Atypical and Malignant Meningiomas. Neurosurgery.

[58] Jenkinson M.D., Javadpour M., Haylock B.J., Young B., Gillard H., Vinten J., Bulbeck H., Das K., Farrell M., Looby S. (2015). The ROAM/EORTC-1308 trial: Radiation versus Observation following surgical resection of Atypical Meningioma: Study protocol for a randomised controlled trial. Trials.

[59] Weber D.C., Schneider R., Goitein G., Koch T., Ares C., Geismar J.H., Schertler A., Bolsi A., Hug E.B. (2012). Spot scanning-based proton therapy for intracranial meningioma: Long-term results from the Paul Scherrer Institute. Int. J. Radiat. Oncol. Biol. Phys..

[60] Weber D.C., Lomax A.J., Rutz H.P., Stadelmann O., Egger E., Timmermann B., Pedroni E.S., Verwey J., Miralbell R., Goitein G. (2004). Spot-scanning proton radiation therapy for recurrent, residual or untreated intracranial meningiomas. Radiother. Oncol..

[61] Murray F.R., Snider J.W., Bolsi A., Lomax A.J., Walser M., Kliebsch U., Schneider R.A., Weber D.C. (2017). Long-Term Clinical Outcomes of Pencil Beam Scanning Proton Therapy for Benign and Non-benign Intracranial Meningiomas. Int. J. Radiat. Oncol. Biol. Phys..

[62] El Shafie R.A., Czech M., Kessel K.A., Habermehl D., Weber D., Rieken S., Bougatf N., Jakel O., Debus J., Combs S.E. (2018). Clinical outcome after particle therapy for meningiomas of the skull base: Toxicity and local control in patients treated with active rasterscanning. Radiat. Oncol..

[63] Slater J.D., Loredo L.N., Chung A., Bush D.A., Patyal B., Johnson W.D., Hsu F.P., Slater J.M. (2012). Fractionated proton radiotherapy for benign cavernous sinus meningiomas. Int. J. Radiat. Oncol. Biol. Phys..

[64] Vlachogiannis P., Gudjonsson O., Montelius A., Grusell E., Isacsson U., Nilsson K., Blomquist E. (2017). Hypofractionated high-energy proton-beam irradiation is an alternative treatment for WHO grade I meningiomas. Acta Neurochir..

[65] Vernimmen F.J., Harris J.K., Wilson J.A., Melvill R., Smit B.J., Slabbert J.P. (2001). Stereotactic proton beam therapy of skull base meningiomas. Int. J. Radiat. Oncol. Biol. Phys..

[66] Halasz L.M., Bussiere M.R., Dennis E.R., Niemierko A., Chapman P.H., Loeffler J.S., Shih H.A. (2011). Proton stereotactic radiosurgery for the treatment of benign meningiomas. Int. J. Radiat. Oncol. Biol. Phys..

[67] Gudjonsson O., Blomquist E., Nyberg G., Pellettieri L., Montelius A., Grusell E., Dahlgren C., Isacsson U., Lilja A., Glimelius B. (1999). Stereotactic irradiation of skull base meningiomas with high energy protons. Acta Neurochir..

[68] Klinger D.R., Flores B.C., Lewis J.J., Hatanpaa K., Choe K., Mickey B., Barnett S. (2015). Atypical Meningiomas: Recurrence, Reoperation, and Radiotherapy. World Neurosurg..

[69] Li H., Zhang Y.S., Zhang G.B., Zhang G.J., Wang B., Li D., Wu Z., Zhang J.T. (2019). Treatment Protocol, Long-Term Follow-Up, and Predictors of Mortality in 302 Cases of Atypical Meningioma. World Neurosurg..

[70] Sahm F., Schrimpf D., Olar A., Koelsche C., Reuss D., Bissel J., Kratz A., Capper D., Schefzyk S., Hielscher T. (2016). TERT Promoter Mutations and Risk of Recurrence in Meningioma. J. Natl. Cancer Inst..

[71] Sahm F., Schrimpf D., Stichel D., Jones D.T.W., Hielscher T., Schefzyk S., Okonechnikov K., Koelsche C., Reuss D.E., Capper D. (2017). DNA methylation-based classification and grading system for meningioma: A multicentre, retrospective analysis. Lancet Oncol..

[72] Youngblood M.W., Miyagishima D.F., Jin L., Gupte T., Li C., Duran D., Montejo J.D., Zhao A., Sheth A., Tyrtova E. (2021). Associations of meningioma molecular subgroup and tumor recurrence. Neuro-Oncol..

[73] Simonetti G., Silvani A., Tramacere I., Farinotti M., Legnani F., Pinzi V., Pollo B., Erbetta A., Gaviani P. (2021). Long term follow up in 183 high grade meningioma: A single institutional experience. Clin. Neurol. Neurosurg..

[74] Corniola M.V. (2022). Why we need new classification models in meningioma management. Acta Neurochir..

[75] Kuranari Y., Tamura R., Tsuda N., Kosugi K., Morimoto Y., Yoshida K., Toda M. (2019). Long-Term Clinical Outcome of First Recurrence Skull Base Meningiomas. J. Clin. Med..

[76] Al-Mefty O., Topsakal C., Pravdenkova S., Sawyer J.R., Harrison M.J. (2004). Radiation-induced meningiomas: Clinical, pathological, cytokinetic, and cytogenetic characteristics. J. Neurosurg..

[77] Couldwell W.T., Cole C.D., Al-Mefty O. (2007). Patterns of skull base meningioma progression after failed radiosurgery. J. Neurosurg..

[78] Borovich B., Doron Y. (1986). Recurrence of intracranial meningiomas: The role played by regional multicentricity. J. Neurosurg..

[79] Borovich B., Doron Y., Braun J., Guilburd J.N., Zaaroor M., Goldsher D., Lemberger A., Gruszkiewicz J., Feinsod M. (1986). Recurrence of intracranial meningiomas: The role played by regional multicentricity. Part 2: Clinical and radiological aspects. J. Neurosurg..

[80] Boto J., Guatta R., Fitsiori A., Hofmeister J., Meling T.R., Vargas M.I. (2021). Is Contrast Medium Really Needed for Follow-up MRI of Untreated Intracranial Meningiomas?. AJNR Am. J. Neuroradiol..

[81] Kanda T., Ishii K., Kawaguchi H., Kitajima K., Takenaka D. (2014). High signal intensity in the dentate nucleus and globus pallidus on unenhanced T1-weighted MR images: Relationship with increasing cumulative dose of a gadolinium-based contrast material. Radiology.

[82] Ueberschaer M., Vettermann F.J., Forbrig R., Unterrainer M., Siller S., Biczok A.M., Thorsteinsdottir J., Cyran C.C., Bartenstein P., Tonn J.C. (2020). Simpson Grade Revisited—Intraoperative Estimation of the Extent of Resection in Meningiomas Versus Postoperative Somatostatin Receptor Positron Emission Tomography/Computed Tomography and Magnetic Resonance Imaging. Neurosurgery.

[83] Gay E., Vuillez J.P., Palombi O., Brard P.Y., Bessou P., Passagia J.G. (2005). Intraoperative and postoperative gamma detection of somatostatin receptors in bone-invasive en plaque meningiomas. Neurosurgery.

[84] Haslund-Vinding J., Skjoth-Rasmussen J., Poulsgaard L., Fugleholm K., Mirian C., Maier A.D., Santarius T., Rom Poulsen F., Meling T., Bartek J.J. (2021). Proposal of a new grading system for meningioma resection: The Copenhagen Protocol. Acta Neurochir..

[85] Afshar-Oromieh A., Giesel F.L., Linhart H.G., Haberkorn U., Haufe S., Combs S.E., Podlesek D., Eisenhut M., Kratochwil C. (2012). Detection of cranial meningiomas: Comparison of (6)(8)Ga-DOTATOC PET/CT and contrast-enhanced MRI. Eur. J. Nucl. Med. Mol. Imaging.

[86] Milker-Zabel S., Zabel-du Bois A., Henze M., Huber P., Schulz-Ertner D., Hoess A., Haberkorn U., Debus J. (2006). Improved target volume definition for fractionated stereotactic radiotherapy in patients with intracranial meningiomas by correlation of CT, MRI, and [68Ga]-DOTATOC-PET. Int. J. Radiat. Oncol. Biol. Phys..

[87] Nassiri F., Mamatjan Y., Suppiah S., Badhiwala J.H., Mansouri S., Karimi S., Saarela O., Poisson L., Gepfner-Tuma I., Schittenhelm J. (2019). DNA methylation profiling to predict recurrence risk in meningioma: Development and validation of a nomogram to optimize clinical management. Neuro-Oncol..

